# Exploring Peltier effect in organic thermoelectric films

**DOI:** 10.1038/s41467-018-05999-4

**Published:** 2018-09-04

**Authors:** Wenlong Jin, Liyao Liu, Tao Yang, Hongguang Shen, Jia Zhu, Wei Xu, Shuzhou Li, Qing Li, Lifeng Chi, Chong-an Di, Daoben Zhu

**Affiliations:** 1 Beijing National Laboratory for Molecular Sciences, CAS Key Laboratory of Organic Solids, Institute of Chemistry, Chinese Academy of Sciences, 100190 Beijing, China; 2 University of Chinese Academy of Sciences, 100049 Beijing, China; 3 Institute of Functional Nano & Soft Materials (FUNSOM), Jiangsu Key Laboratory for Carbon-Based Functional Materials & Devices, Soochow University, 215123 Suzhou, China; 4 Key Laboratory of Theoretical and Computational Photochemistry, Ministry of Education, College of Chemistry, Beijing Normal University, 100875 Beijing, China; 5 Center for Programmable Materials, School of Materials Science and Engineering, Nanyang Technological University, 50 Nanyang Avenue, 639798 Singapore, Singapore

## Abstract

Organic materials are emerging thermoelectric candidates for flexible power generation and solid-cooling applications. Although the Peltier effect is a fundamental thermoelectric effect that enables site-specific and on-demand cooling applications, the Peltier effect in organic thermoelectric films have not been investigated. Here we experimentally observed and quasi-quantitatively evaluated the Peltier effect in a poly(Ni-ett) film through the fabrication of thermally suspended devices combined with an infrared imaging technique. The experimental and simulation results confirm effective extraction of the Peltier effect and verify the Thomson relations in organic materials. More importantly, the working device based on poly(Ni-ett) film yields maximum temperature differences as large as 41 K at the two contacts and a cooling of 0.2 K even under heat-insulated condition. This exploration of the Peltier effect in organic thermoelectric films predicts that organic materials hold the ultimate potential to enable flexible solid-cooling applications.

## Abstract (web summary)

Observing the Peltier effect, e.g. cooling/heating at material junctions due to current flow, in organic thermoelectric films remains a challenge due the inherent properties of these materials. Here, the authors use IR imaging to experimentally observe the Peltier effect in poly(Ni-ett)-based films.

## Introduction

Thermoelectric (TE) materials allow direct conversion between heat and electricity via the Seebeck and Peltier effects, making their devices reliable power generators and solid-cooling elements without moving parts^1^. Recent extensive research into the Seebeck effect of organic materials has led to *ZT* values >0.2 and enables them competitive candidates for flexible TE applications^2–7^. The Peltier effect occurs when electrical current flows across an isothermal junction of two materials, leading to a cooling/heating effect at the contact depending on the direction of current flow and the sign of the Peltier coefficient^8,9^. Experimentally probing the Peltier effect in TE materials is crucial not only to understand their charge transport properties and charge–phonon interactions^10–12^ but is also critical for demonstrating their solid-state-cooling applications^13–20^. Despite the fundamental significance and recent exciting observation of Peltier effect in junctions at molecular scale,^2,10,11^ potential cooling ability of organic TE (OTE) film promised by their remarkable performance at room temperature has not yet been demonstrated owing to the unrevealed Peltier effect in organic thin-film devices.

Peltier effect occurs in a TE device concurrently with several heat generation and transfer processes, including the Joule heating, internal heat conduction, and various heat dissipation processes (interlayer heat conduction, convection, and radiation) (Supplementary Fig. 1), making studies on Peltier effect a complicated issue. Precise extraction of the Peltier effect in OTE materials is particularly challenging because of the large contribution of Joule heating caused by the relative low electrical conductivity of OTE materials^3–7,21^, and significant effect of heat dissipation on the measured temperature distribution result from their relative low thermal conductivity. More importantly, the highly anisotropic performance (*ZT*_⫽_ ≫*ZT*_⊥_) in OTE films impedes investigations of their Peltier effect in the dominant charge transport direction using bulk materials with sandwiching device architecture. Investigation of Peltier effect in OTE materials is, therefore, limited by the precise identification of Peltier cooling ability among the complicated thermal processes in a lateral thin-film device.

Here we use a thermally suspended device and an infrared (IR) imaging technique to probe the Peltier effect in an organic film based on poly(Ni-ett). Our results not only reveal the effect of Peltier cooling at the contacts but also enable extraction of the Peltier coefficient to validate the Thomson relation in OTE materials. These quasi-quantitative observations, combined with the cooling ability demonstrated by experiments and simulations, indicate promising applications of OTE materials in future cooling elements.

## Results

### Basic mechanism and device optimization

Poly(Ni-ett) is used as an OTE material because of its prominent TE performance and excellent stability^21^. The involved thermal processes of a conventional thin-film device with lateral geometry, which is composed by two gold electrodes and the poly(Ni-ett) film, are depicted in Supplementary Fig. 1. With the current directed from the poly(Ni-ett) film to the gold electrode, the Peltier cooling power at the contact is determined by the equation d*Q*/d*t* = (*Π*_Ni-ett_ − *Π*_Au_)*I* ≈ *Π*_TE_*I* because |*Π*_Ni-ett_| ≫ *Π*_Au_, where *Π* and *I* are Peltier coefficient and current, respectively. Reversely, another contact where the current flows from gold to poly(Ni-ett) has the same heating capability. Along with the Peltier effect, many aforementioned thermal processes also co-exist in an operating device and play a completing role in determining the distribution of temperature. For instance, the Joule heating and various heat dissipation processes contribute to the increased and decreased temperature of the device, respectively. For a poly(Ni-ett)-based device on a glass substrate, a combination of multiple effects results in an unobvious Δ*T* < 50 mK at the two contacts even under a current density of 1.5 A mm^−2^ (Supplementary Fig. 2-3 and Supplementary Note 1). Given that the thermal radiation can be neglected, heat dissipation occurs “vertically” into the substrates (interlayer heat conduction) and the air (heat convection) dominates the temperature distribution in the film, leading to the nearly unobserved Peltier effect in a conventional thin-film device. The result indicates that the relative low thermal conductivity of OTE materials make their temperature distribution susceptible against external atmosphere. Consequently, the detection of Peltier effect in OTE films is challenging and the construction of heat-insulated devices are extremely important for studying the Peltier effect in OTE thin films.

**Fig. 1:**
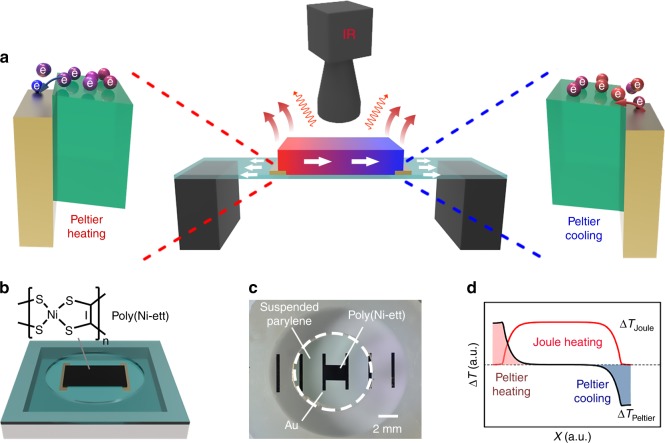
Mechanism of Peltier effect and device geometry. **a** A schematic illustration of the Peltier effect and other involved thermal processes in a thin-film thermoelectric (TE) device with lateral structure. Peltier effect, Joule heating, internal heat transfer within TE film, interlayer heat conduction to the substrate, heat convection to the air, and thermal radiation occurs concurrently in the device. **b** A schematic illustration of the organic TE (OTE) device on the suspended parylene film and the molecular structure of poly(Ni-ett). **c** Photograph of an OTE device on a 300 nm suspended parylene film. **d** A schematic illustration of the temperature distribution caused by Joule heating and by the Peltier effect. Electrical cooling and heating at the different electrode/TE contacts enable the device to function as a heat engine for solid-state cooling elements

We investigated the Peltier effect by fabricating a poly(Ni-ett)-based device on an ultrathin (300 nm) suspended parylene film (Fig. 1a). Parylene is used as the heat-insulation substrate because of its low thermal conductivity and excellent mechanical properties (*κ*_RT_ = 0.084 W m^−1^ K^−1^, Young’s modulus: 2.8 GPa). The fabrication information is detailed in Methods and Supporting Information sections (Fig. 1 and Supplementary Fig. 4). The poly(Ni-ett) film exhibits a *ZT*_300K_ of 0.06 because of multiple transfer processes during device fabrication (Supplementary Fig. 5). Compared with glass-substrate-based devices measured in air, the film was measured using a thermally suspended device geometry and maintaining a vacuum of 6 × 10^−4^ Pa to minimize the heat dissipation, as indicated by the dramatically increased Δ*T* (>200 times) at the two contacts from <0.05 K to >12 K under a current density of 1.5 A mm^−2^ (Supplementary Fig. 6-8). The result highlights the thermally suspended feature of the devices.

### Observation of the Peltier effect in OTE film

**Fig. 2:**
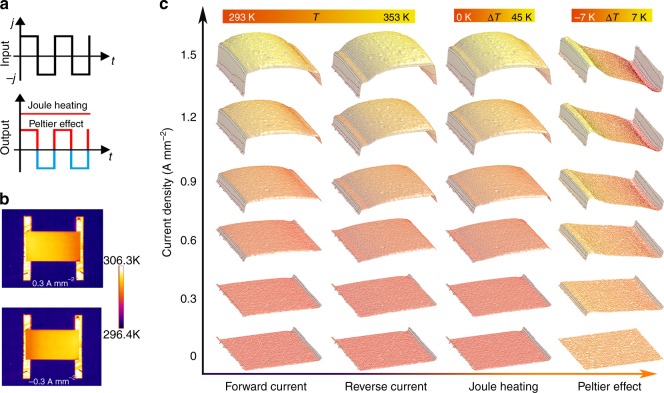
Imaging of the Joule heating and Peltier effects of an organic thermoelectric (OTE) device. **a** A schematic illustration of the mechanism for separating Joule heating and the Peltier effect. The Peltier effect and Joule heating are odd and even functions of the applied current, respectively. When a rectangular alternating current is applied, the direction of the temperature distribution induced by the Peltier effect changes with the current, whereas the Joule-heating-induced temperature distribution remains constant. **b** Infrared image of an OTE device with a current of ±0.3 A mm^−2^. Current flow from the left electrode to the right electrode is defined as the forward direction (+) and vice versa (−). **c** Temperature distribution of an OTE device under different currents and the separate contributions of Joule heating and the Peltier effect to the temperature of the film. The sharp temperature drop of the exposed electrodes arises from low emissivity of gold electrodes

Having fabricated a thermally suspended OTE device, we use it to study the spatially resolved temperature distribution with IR microscopy (Supplementary Fig. 9). In this case, the Peltier effect and Joule heating can be distinguished in a single measurement scan^22–24^, since the former effect is a reversible thermodynamic phenomenon that depends linearly on the current (∝*I*) and matches the bias frequency, whereas Joule heating is fundamentally different and exhibits irreversible quadratic response (∝*I*^2^) (Fig. 2a). Consequently, we obtained the contribution from the Peltier effect to the temperature distribution by analyzing the signal response under alternating current bias with rectangular wave modulation $\Delta T_{{\mathrm{Peltier}}}{\mathrm{ = }}(\Delta T_{{j} + } - \Delta T_{{j} - })/2,\Delta T_{{\mathrm{Joule}}} = (\Delta T_{{j} + } + \Delta T_{{j} - })/2$.

Figure 2b compares the recorded temperature distribution of a working device under current flow of ±0.3 A mm^−2^. Joule heating is confirmed by the increase in temperature of the entire device, whereas the Peltier effect is evidenced by the reversed temperature distribution along with switched current direction. These combined effects lead to an asymmetric increase in temperature and to a linear-like temperature profile depending on the current direction. Figure 2c further illustrates the spatially resolved temperature distribution of the device and the individual contributions of two effects under different current biases. When the current density is <0.9 A mm^−2^, uniform Joule heating across the film contributes to around one half of the temperature change. In contrast, Joule heating at a current density of 1.5 A mm^−2^ accounts for more than four fifths of the temperature change at the contact regime, showing that the temperature rise scales with the square of the biasing current. It should be noted that the heat sink effect of the electrodes becomes substantial with an increase in current density, leading to a nonuniform contribution from Joule heating near the contacts. By contrast, the temperature distribution induced by the Peltier effect is centrosymmetric with a vanishing temperature change in the center, regardless of the currents (Fig. 2c). Notably, the maximum Δ*T* at the two contacts increases linearly from 2.8 to 12 K, along with an increase in current density from 0.3 to 1.5 A mm^−2^. This result is consistent with a proportional relationship between the Peltier effect and the magnitude of the current, which further verifies the direct observation of the Peltier effect in an OTE film.

### Transient IR measurement

**Fig. 3:**
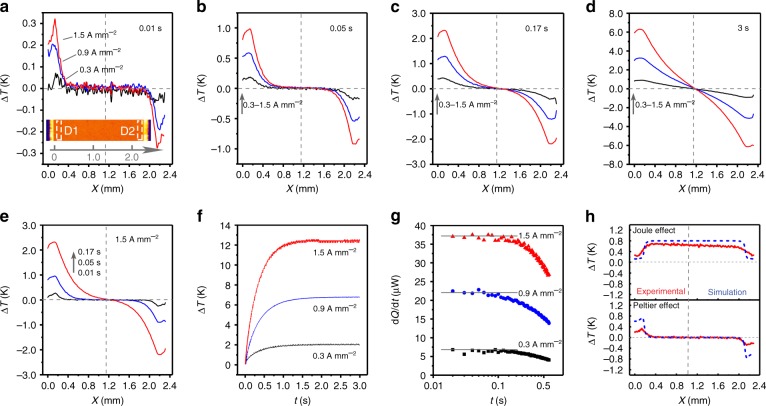
Transient infrared characterization of Peltier effect. Peltier-effect-induced temperature distribution in the current direction at current densities of 0.3, 0.9, and 1.5 A mm^−2^ for **a** 0.01 s, **b** 0.05 s, **c** 0.17 s, and **d** 1 s. **e** Peltier-effect-induced temperature distribution in the current direction at 0.01, 0.05, and 0.1 s with a current density of 1.5 A mm^−2^. **f** Temperature difference at the two contacts versus biasing time for different current densities. **g** Measured Peltier heating/cooling power with respect to time. **h** Experimental and simulated temperature distribution contributed by the Joule heating and Peltier effect under a current density of 1.5 A mm^−2^ for 0.01 s

To explore how the Peltier effect contributes to the temperature change of the OTE film, we performed transient IR characterization with a frequency of 500 Hz. Figures 3a–d illustrates the biasing-time-dependent contribution of the Peltier effect to the temperature change of the film under different current bias. Benefiting from rapid heating/cooling feature of the Peltier effect, an intermediate current density of 0.3 A mm^−2^ leads to a slight change in temperature (<0.1 K) near the contacts within 10 ms (Fig. 3a). An increase in the current density from 0.3 to 1.5 A mm^−2^ results in linearly enhanced changes in temperature from 0.08 to 0.34 K. Of particular note, Peltier cooling/heating is confined to areas near the contacts (<300 µm) within 10 ms. A longer operating time contributes to an obvious increase in Δ*T* and to an enhanced distance of heat diffusion (Fig. 3b–e). The heating/cooling energy diffuses into the central part of the film at 0.17 s, where the Δ*T* at the two contacts reaches 4.6 K. Thereafter, a thermal balance caused by the Peltier effect and internal heat conduction across the film can be achieved within a few seconds regardless of the current density. As an example, the Δ*T* at the two contacts shows a saturation at 12.6 K upon a current density of 1.5 A mm^−2^ for <3 s. In one word, the competition between the rapid Peltier heating/cooling and relative slow internal heat conduction leads the nonlinear increase in the maximum Δ*T* with time (Fig. 3f). Figure 3g further presents the change in the measured Peltier heating/cooling power (d*Q*/d*t*) with respect to time. It should be noted that the measured power is nearly independent of the biasing time within 0.2 s. Continued biasing results in an obvious decrease in measured power due to the aforementioned heat conduction within the film. Consequently, the investigation of the transient temperature distribution with a biasing time <0.2 s is vital to understanding the dynamic contribution processes from Peltier effect in this thin-film device.

Finite element simulation of the transient temperature distribution (0.01−0.1 s) in an ideally adiabatic device (without any substrates and contacting probes) was performed to testify our experimental results (see Supplementary Figs. 10-11 and Supplementary Note 2). When a device operates at a current bias of 1.5 A mm^−2^ for 10 ms, the simulated Joule heating yields a uniform temperature increase of 0.80 K, which is higher than that of the experimental results (0.62 K). This slight difference is mainly caused by the weak heat dissipation to the ultrathin suspended parylene film. In contrast, the experimental result of Peltier effect (Δ*T*_max_ = 0.34 K, *Q*_p_ = 0.58 µJ) corresponded to 45.3%/50.9% of the predicted values in an ideal device (Δ*T*_max,s_ = 0.75 K, *Q*_p,s_ = 1.14 µJ) (Supplementary Fig. 11). The relative large difference relies on the intrinsic feature that the Peltier effect only occurs at the Au/poly(Ni-ett) contact, leading to particular large effect of unavoidable heat dissipation (to the contacting probes through electrodes) to the measured cooling/heating power at the two contacts. Notably, the Peltier effect in poly(Ni-ett) films can be hardly observed in conventional devices (<0.2% of predicted result), and the thermally suspended device thus enables quasi-quantitative evaluation of Peltier effect in OTE films.

## Discussion

The Peltier effect in TE material is typically quantified by *Π*, which can be used to describe the heat transported by thermally excited charge carriers^11^. However, the experimental determination of *Π* in a thin film is extremely difficult because the overall heat flux and thermal diffusion flux must be measured quantitatively and simultaneously in an isothermal condition^9^. Lock-in IR measurement can simulate a quasi-isothermal condition and provides an opportunity to extract *Π* using the method proposed by Breitenstein^25^. *Π* can be expressed as

$${\Pi}_{{\mathrm{Poly}}\left( {{\mathrm{Ni}} - {\mathrm{ett}}} \right)} - {\Pi}_{{\mathrm{Au}}} \approx {\Pi}_{{\mathrm{Poly}}\left( {{\mathrm{Ni}} - {\mathrm{ett}}} \right)} = - U{\int} {Q^{\mathrm{P}}{\mathrm{d}}x/{\int} {Q^{\mathrm{J}}{\mathrm{d}}x} }$$

where *Π*_poly(Ni-ett)_ and *Π*_Au_ are the Peltier coefficient of poly(Ni-ett) and the Au electrode, respectively. *U* is the voltage decrease across the device. *Q*^P^ and *Q*^J^ are the energies of Peltier cooling/heating and the Joule effect, respectively. By experimentally determining *Q*^P^, *Q*^J^, and *U* with lock-in IR technique (see Supplementary Fig. 12 and Supplementary Note 3), we obtain a *Π* of −21.6 mV at room temperature. This value is consistent with the results (−23.5 mV) predicted by the Thomson relation (*Π* = *ST*, where *S* is Seebeck coefficient, *S*_298K_ = −79 µV K^−1^), providing an experimental evidence that the Thomson relation is applicable for OTE materials.

**Fig. 4:**
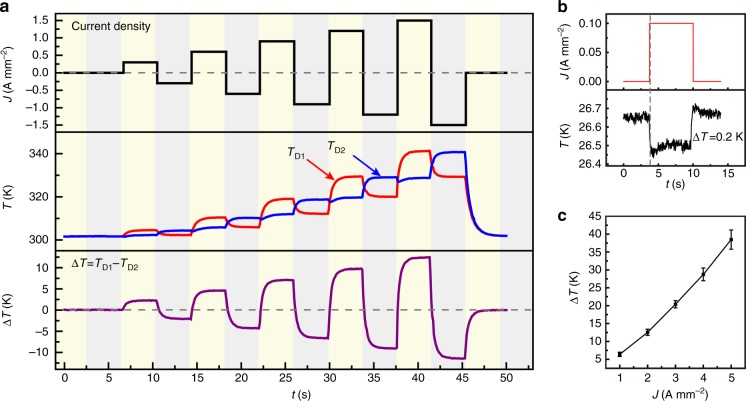
Real-time characterization of the temperature differences. **a** Real-time temperature of the two contacts in an organic thermoelectric (OTE) device and the extracted temperature differences at different current densities. The thermal equilibrium is established within 3 s. **b** Peltier-effect-induced temperature at the cooling contact of an OTE device under a current density of 0.1 A mm^−2^. **c** Current-density-dependent temperature differences at the two contacts of the device. A large temperature difference of up to 41 K is observed at the current density of 5 A mm^−2^

To demonstrate the ability to generate Δ*T* across an OTE device, we performed real-time measurements under different current biases (Fig. 4). As current density increased, the temperature of the two contacts rise simultaneously and a saturated Δ*T* is rapidly established (Fig. 4a, Supplementary Movie 1). Interestingly, a large Δ*T* of 38 ± 3 K under a current density of 5 A mm^−2^ is observed. More importantly, a cooling of one contact by 0.2 K is observed under a current density of 0.1 A mm^−2^ even if the device operates under heat-insulated condition, demonstrating the promising cooling ability of the poly(Ni-ett)-based film. A much superior realistic cooling ability is feasible in future OTE device, for which we expect better TE performance, improved contacts and more reasonable device geometry. This deduction is confirmed by the simulation results (Supplementary Fig. 13-14 and Supplementary Note 4), from which an effective cooling of 25 K and a high heat-flux pumping capacity >600 W cm^−2^ are predicted if ultrathin (<10 µm) vertical OTE devices can be fabricated with reported in-plane performance (*ZT* > 0.2)^3–6,21^, ideal ohmic contacts, and an optimized interfacial thermal resistance^16,26^.

In conclusion, we have experimentally observed and quasi-quantitatively studied the Peltier effect in poly(Ni-ett)-based films. The Peltier coefficient was also measured to confirm the Thomson relation in conducting polymer. Furthermore, the large Δ*T* observed at the two contacts together with an obvious cooling behavior under heat-insulated condition and the potential cooling ability predicted by the simulation, suggests that OTE materials are promising cooling candidates. Our work is expected to stimulate further systematic studies of the different underlying physical processes in OTE materials and the construction of Peltier cooling devices using emerging organic materials.

## Methods

### Growth of poly(Ni-ett) film

Poly(Ni-ett) was polymerized by an electrochemical method in a glove box. The precursor was prepared according to the literature^21^. Electrochemical polymerization was performed in a one-compartment cell in a glove box. All experimental set-ups were as same as those previously reported. The cleaned polyethylene terephthalate substrates were connected to the working electrodes, which were carefully polished and successively cleaned with water and acetone. The poly(Ni-ett) film was obtained by potentiostatic anodic oxidation of the precursor solution. The potential was maintained at 0.6 V for 28 h.

### Device fabrication

Corning glass was sequentially cleaned with deionized water, ethanol, and acetone. After being dried in a nitrogen flow, the substrates were modified with a self-assembled octadecyltrichlorosilane (Sigma-Aldrich) monolayer at 120 °C in vacuum. A 300 nm-thick parylene film was then deposited onto the modified substrate by using PDS2010 (SCS Labcoter) system. Parallel-patterned electrodes (Ti/Au, 5/95 nm) were then thermally deposited onto the parylene using a shadow mask. The length and width of the electrodes and the distances between them were 4000, 300, and 2000 μm, respectively.

The poly(Ni-ett) film was immersed in deionized water and methanol for 3 h. The film (2 × 1.5 mm^2^) was then detached and transferred to the substrate. The device was annealed under vacuum at 100 °C overnight. The thermally suspended thin-film devices were obtained by peeling off the glass substrate, leaving the device fixed to the holder and ready for testing.

### Device measurement

The temperature distribution was measured using a high-speed IR camera (FLIR X6530sc, an IR camera with a temperature sensitivity of 20 mK and a spatial resolution of 15 µm) in a homemade high-vacuum (6 × 10^−4^ Pa) chamber with a germanium IR window (Edmund Optics, light transmittance >95% in the IR range of the window). The devices were connected to the measurement system using gold wire (25 µm in diameter) to minimize the heat dissipation. The electrical conductivity and Seebeck coefficient were measured by a standard 4-probe method with a Keithley 4200 and SB100, respectively. The thickness of the film was measured using a Dektak XT stylus profiler.

## Electronic supplementary material

Supplementary Information

Peer Review File

Description of Additional Supplementary Files

Supplementary Movie 1

## Data Availability

The data that support the findings of this study are available from the corresponding authors upon request.
