# Supplementary material for: Exploring Peltier effect in organic thermoelectric films
Source: Nat Commun. 2018 Sep 4;9:3586. doi: 10.1038/s41467-018-05999-4 (PMC6123419; doi:10.1038/s41467-018-05999-4)
Supplement: Supplementary file 1 — Supplementary Information [file 41467_2018_5999_MOESM1_ESM.pdf]

Supplementary Information

Exploring Peltier Effect in Organic Thermoelectric Films

Jin et al.

Supplementary Notes 1 | Temperature calibration

To obtain the temperature distribution of a poly(Ni-ett)-based film, we measured the relationship between T_{IR} (measured by IR camera) and T_{PT100} (measured by a PT100 temperature sensor). All influence factors caused by the camera, IR window, and the properties of film were included in the calibration parameter. The poly(Ni-ett) film for temperature calibration was utilized without further treatment. The calibration was as follows:

(1) Set the default parameters of the IR camera.

(2) Measure the temperature of poly(Ni-ett) in steady-state using the IR camera and PT100 temperature sensor.

(3) Fit T_{IR} and T_{PT100} to obtain the working curve.

(4) Calibrate the temperature of the tested device pixel by pixel.

The calibration was performed in the same apparatus and testing environment (6×10^{-4} Pa). All data were fitted by a concatenate fitting mode because of the favorable repeatability of the five tested devices. After calibration, the change in temperature induced by the biased current was measured using the FLIR X6530sc directly. The result are comparable to those obtained using the PT100 temperature sensor.

Supplementary Notes 2 | Finite element simulation of lateral thin-film device

To testify the transient temperature distribution of the device, we simulated the transient work condition of the device using thermoelectric module of the COMSOL Multiphysics software. The simulation results based on the model and boundary conditions are described below.

The poly(Ni-ett) was set as a cuboid (2.3 mm×1.5 mm×2.15 μm). Two Au electrodes (1500 μm×150 μm× 95 nm) were placed above the conducting material on two sides of the sample. All transient simulation results below are based on this device geometry. The model is schematically illustrated in Figure S9a.

In our simulation system, the Au/poly(Ni-ett) interface was assumed to have idea thermal contacts. Interfacial thermal conductance between the device and the atmosphere was omitted because the test was performed under 6×10^{-4} Pa. The initial temperature condition was set as constant (T_0 = 298.15 K). All surfaces of the model are radiating active. In simulations, various currents were applied to the left gold electrode, whereas another gold electrode was grounded.

All physical properties of gold were based on built-in parameters. The temperature-dependent Seebeck coefficient and electrical conductivity of poly(Ni-ett) film were measured by SB100 and Keithley 4200SC, respectively. The heat specific heat capacity was measured using differential scanning calorimeter (TA Q2000 with compacted block sample). All relevant parameters of the materials are summarized in Supplementary Table 1 and Supplementary Figure 2.

Supplementary Notes 3 | Lock-in IR measurement

Lock-in thermography can overcome the limitations of temperature resolution associated with the IR camera by long-term data acquisition and data processing. Here, we used this technique to distinguish the Peltier effect and Joule heating of poly(Ni-ett) film in small temperature modulation.

The temperature modulation of the OTE device was caused by the combined influence of the Peltier effect and Joule heating. When an a.c. current was applied to the device, the temperature modulations caused by these two effects are the first and second harmonic signals, respectively. Therefore, the extracted amplitudes of first and second harmonic represented the temperature changes caused by the Peltier effect and Joule heating, which can be used to calculate the Peltier coefficient.

In our experiments, two synchronous cosine currents were simultaneously applied to the device and IR camera. The frequency of the applied current was selected to limit the thermal diffusion length of the heat wave1. The detected temperature of the device was almost unchanged during the experiment duo to the very low driving a.c. current. After data processing, the first and second harmonic signal was both substantial. The extracted first harmonic signal represented the Peltier temperature modulation and was limited near the two electrode/poly(Ni-ett) interfaces, consistent with the fact that the Peltier effect is an interface effect. In addition, the phase difference of these two signals is 180°, indicating that one electrode/poly(Ni-ett) contact is cooled while another one is heated. The extracted second harmonic signal is uniform in the entire device, implying that the Joule heating is uniform in the entire device without any phase difference[1,2].

Supplementary Notes 4 | Finite element modeling simulation of ultrathin vertical device

We simulated a single leg ultrathin device to predict the Peltier cooling ability of poly(Ni-ett). The cross-section of the device was set as a square (1 ×1 mm^2) and the thickness of the organic material was varied from 2 to 14 μm. All TE parameters of the poly(Ni-ett) (the maximum performance in literature)[3] are listed in Supplementary Table 2.

In our model, all thermal contacts and electrical contacts were assumed to be ideal. The upper ceramic functioned as cooling surface and the bottom one worked as heat sink with maintained temperature of 300 K. In addition, all interfaces were regarded as radiating active. In the simulation, the ΔT and heat transport capacity were evaluated by applying various current densities (10−100 Amm^{-2}) to the upper gold electrode, whereas the bottom electrode was grounded. The simulated cold side temperature and temperature difference between two ceramic slices based on this thermoelectric material is shown in Figure S13b and S13c.

For the cooling device, the optimal currents ($I_{\mathrm{opt}} = ST_{\mathrm{c}}/R$) for the maximum ΔT ($\Delta T_{\mathrm{max}} = ZT^2/2$), varies depending on TE performance of the device[4]. The ΔT increases with increasing current and reaches the maximum ΔT at I_{opt}. However, a further increase in the current degrades the ΔT because of rapidly increased Joule heating.

Transported heat flux, an important parameter to characterize the performance of Peltier cooling device, is given by

$$q_{\mathrm{max}} = \frac{1}{l}\left\{\frac{S^2 T_{\mathrm{cold-side}}^2 \sigma}{2} - \kappa\left(T_{\mathrm{hot-side}} - T_{\mathrm{cold-side}}\right)\right\}$$

where l is the thickness of the thermoelectric material, S the Seebeck coefficient, σ is the electrical conductivity, and κ is the thermal conductivity. Theoretically, it is

favorable for a thin-film OTE cooling device to have a high heat flux because of its intrinsically low thermal conductivity. Figure S14d shows the heat transport capacity of poly(Ni-ett) ultrathin device, showing the promising Peltier cooling ability of ultrathin OTE materials.

To specify the TE cooling ability of the OTE material, we define a parameter $r = \Delta T_{\mathrm{Poly(Ni-ett)}} / \Delta T_{\mathrm{Bi_2Te_3}}$ to demonstrate the cooling capacity of poly(Ni-ett) (see Supplementary Figure S14). The advantageous regime of poly(Ni-ett) is highlighted by the cyan square. The thinner the TE material, the more the significant relative advantage in ΔT is maintained by the ability of poly(Ni-ett). This result illustrates that the OTE materials are promising candidates for ultrathin Peltier cooling applications.

Supplementary Table 1 Physical properties of materials relevant for simulation.

	Symbol/Unit	Gold	Poly(Ni-ett)[a]
Thermal conductivity	λ [$\mathrm{W\ m^{-1}\ K^{-1}}$]	317	0.5
Electric conductivity	σ [$\mathrm{S\ m^{-1}}$]	4.56×10^{7}	1.4×10^{4}
Density	ρ [kg m^{-3}]	8920	2160
Heat capacity	C [J kg^{-1} K^{-1}]	129	$Cp_1(T)$
Relative permittivity	ε_r	1	1
Seebeck Coefficient	S [$\mathrm{V\ K^{-1}}$]	6.5e-6	$S_1(T)$

Poly(Ni-ett)[a], thermoelectric parameters of transferred film.

Supplementary Table 2 Physical properties for ultrathin device simulation.

	Symbol/Unit	Poly(Ni-ett)[b]
Thermal conductivity	λ [$\mathrm{W\ m^{-1}\ K^{-1}}$]	0.5
Electric conductivity	σ [$\mathrm{S\ m^{-1}}$]	2.0×10^{4}
Density	ρ [kg m^{-3}]	2160
Heat capacity	C [J kg^{-1} K^{-1}]	$Cp_1(T)$
Relative permittivity	ε_r	1
Seebeck Coefficient	S [$\mathrm{V\ K^{-1}}$]	$S_2(T)$

Poly(Ni-ett)[b], Reported thermoelectric parameters of poly(Ni-ett) in Ref 3.

Supplementary Figures

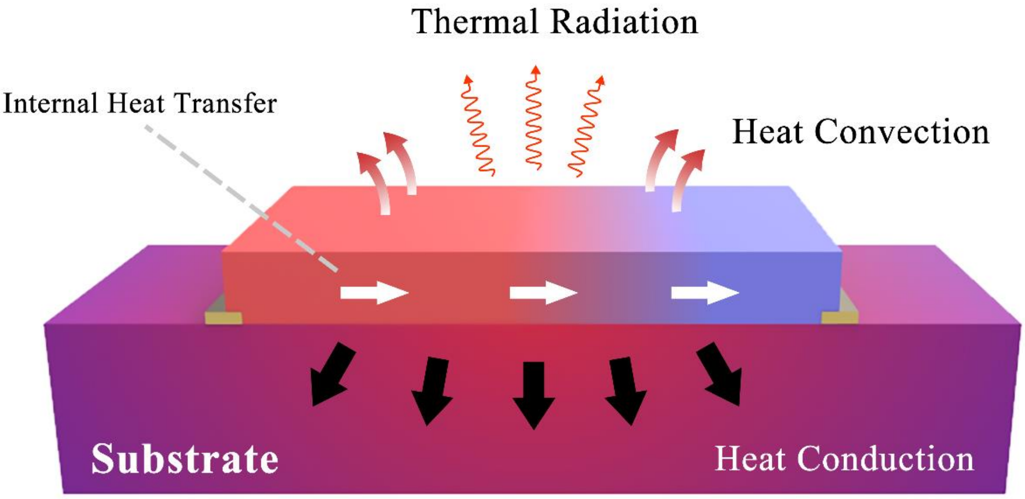

Supplementary Figure 1 | Thermal processes in a conventional TE device with lateral device geometry. Peltier effect, Joule-heating, internal heat transfer within TE film, interlayer heat conduction to the substrate, heat convection to the air, and thermal radiation occurs concurrently in the device. Notably, heat dissipation occurs "vertically" into the substrates (interlayer heat conduction) and the air (heat convection) dominates the temperature distribution in the film, leading to the nearly unobserved Peltier effect in a conventional thin-film device.

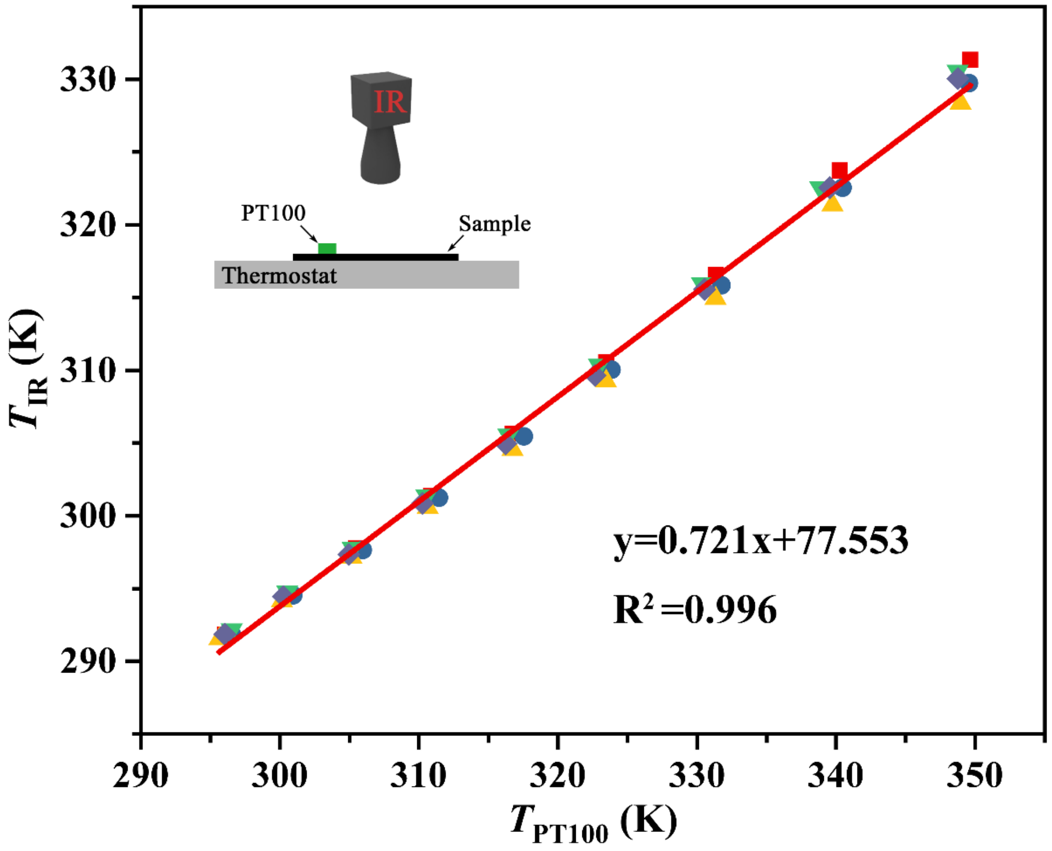

Supplementary Figure 2 | Temperature calibration. Dependence of temperature measured by FLIR X6530sc to the temperature measured by PT100. The data are linear fitting in one line by concatenate fitting mode. All the temperature data is calibrated accordingly. The inset schematically illustrates the setup for temperature calibration.

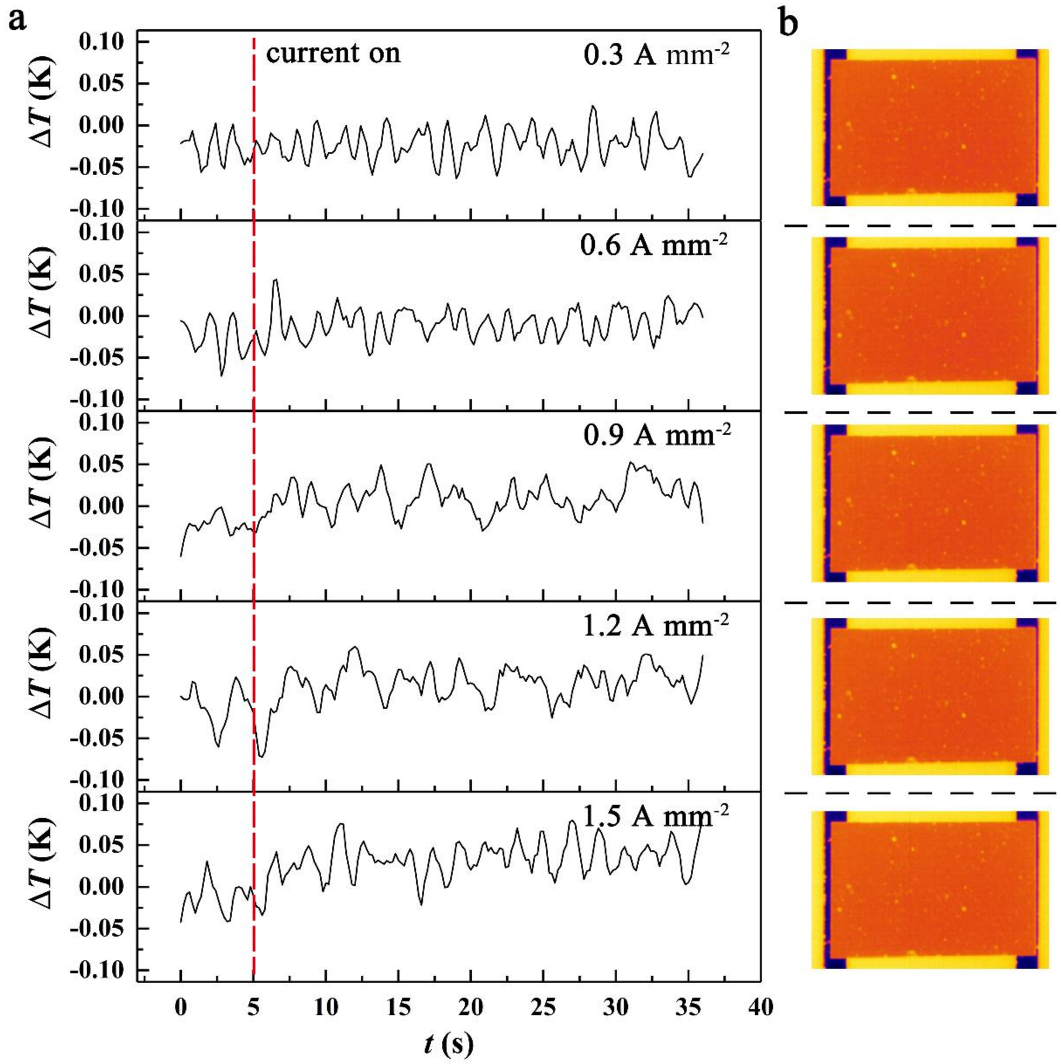

Supplementary Figure 3 | a, Time dependent temperature difference at the two contacts of poly(Ni-ett) devices based on glass substrates at various current densities. **b,** IR image of the working device at different current densities. The temperature of the exposed electrodes is inaccurate arises from low emissivity of gold electrodes.

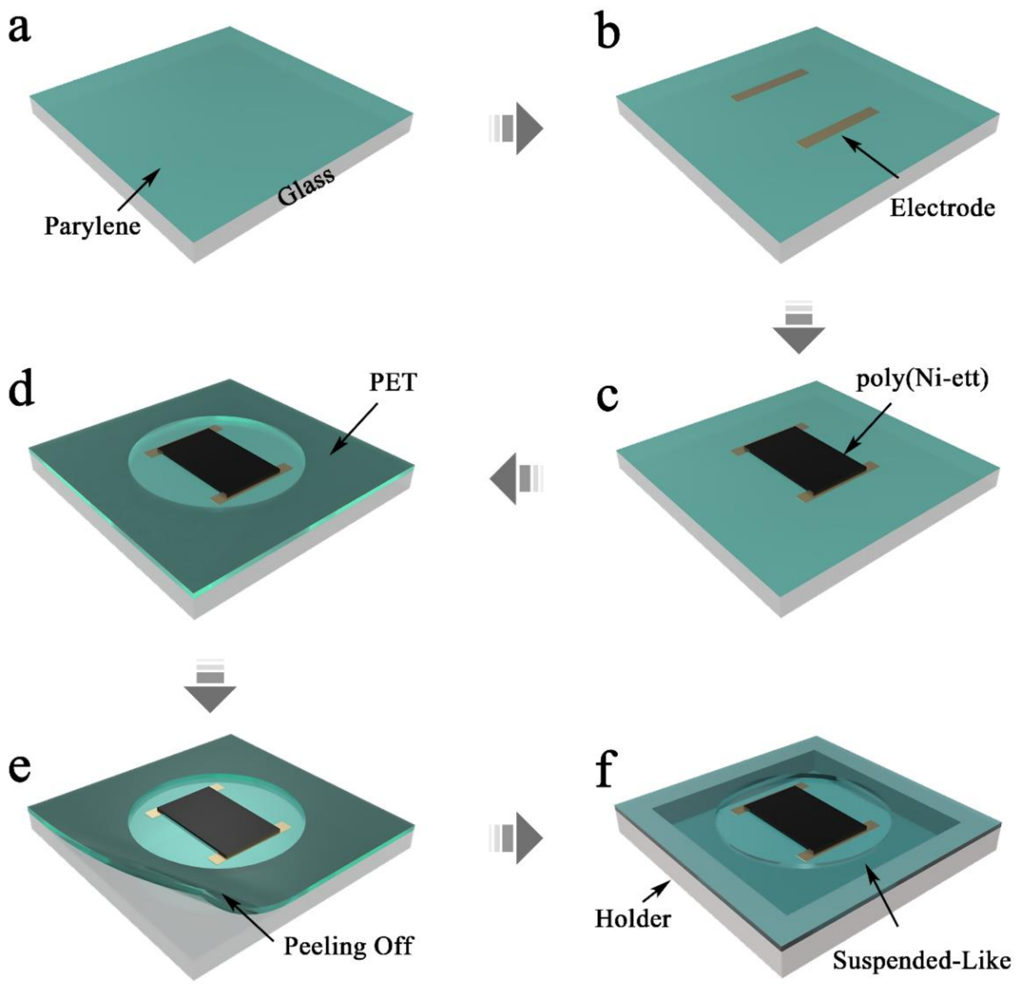

Supplementary Figure 4 | Fabrication procedures of the device. a, Deposition of Parylene film (300 nm) on an OTS modified glass substrate. **b,** Deposition of Ti/Au electrodes onto the Parylene film. **c,** Transferring poly(Ni-ett) to the Parylene substrate and drying under vacuum. **d,** Pasting perforated double-side adhesive and PET to the upper side of the device. **e,** Peeling off the thermal-suspended device (Parylene substrate, gold electrodes and poly(Ni-ett) film) from the glass substrate. **f,** With the device supported on the holder, the thermal-suspended device is ready for testing.

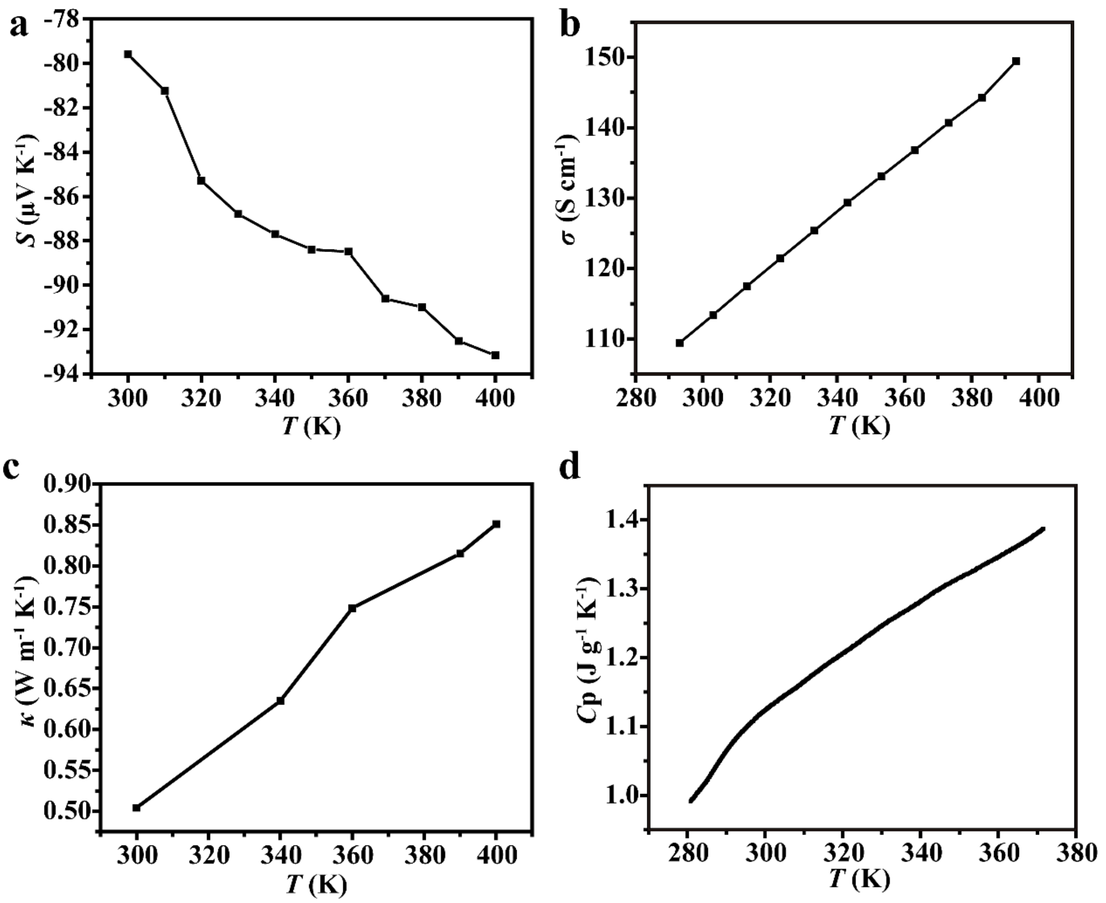

Supplementary Figure 5 | Temperature dependent-thermoelectric performance of thermal-suspended film. Temperature-dependent **a,** Seebeck coefficient, **b,** Electrical conductivity, **c,** Thermal conductivity, and **d,** Specific heat capacity of poly(Ni-ett).

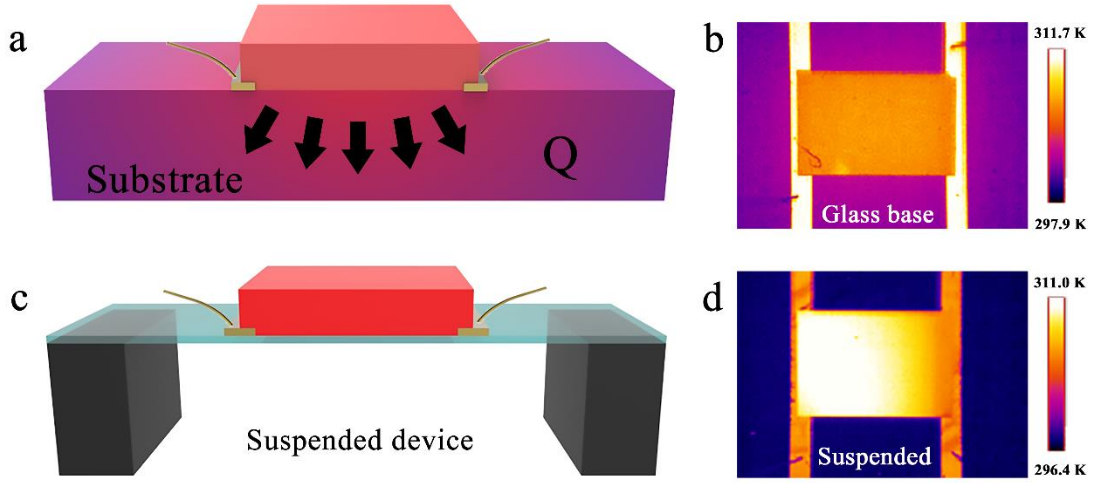

Supplementary Figure 6 | The influence of device structure on temperature distribution. a, Schematic of a device based on glass substrate. **b,** IR image of glass substrate-based device, the temperature difference at the two contacts can be hardly observed due to significant heat exchange between poly(Ni-ett) film and glass substrate. **c**, Schematic of thermal-suspended device fabricated on Parylene substrate. **d,** The IR image of thermal-suspended device. Heat dissipation to Parylene is negligible indicated by the obvious temperature difference at the two contacts and maintained temperature of the Parylene substrate.

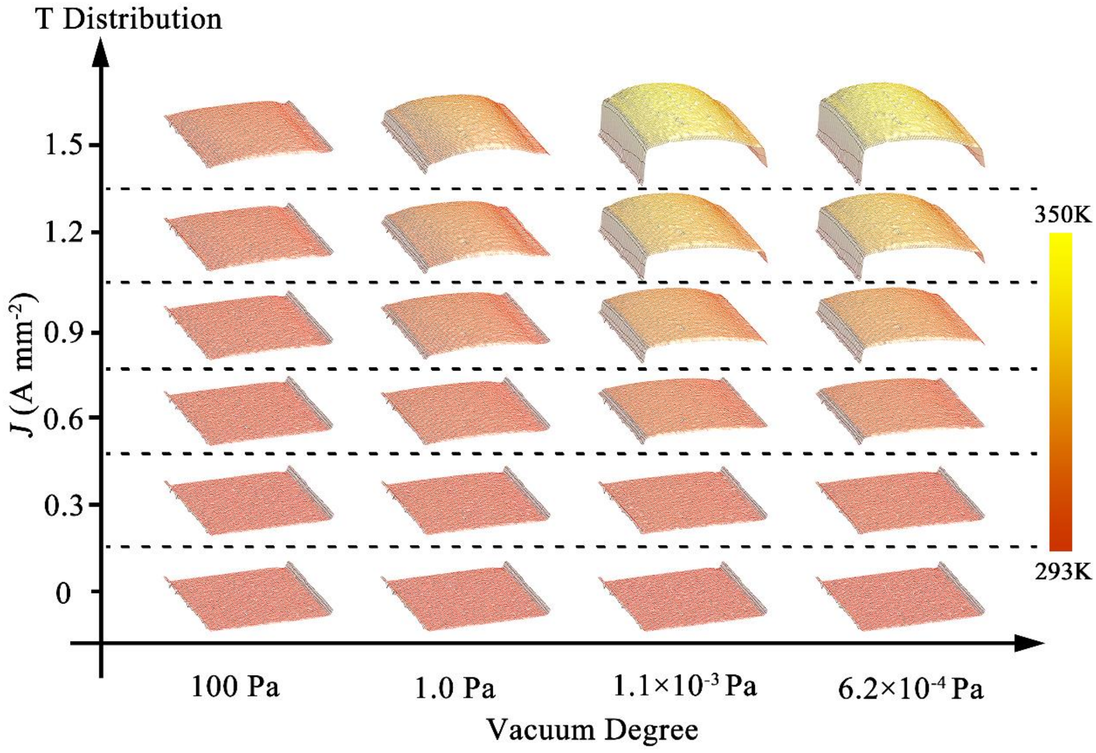

Supplementary Figure 7 | Temperature distribution of an entire device measured at various vacuum and current densities. The sharp temperature drop of the exposed electrodes arises from low emissivity of gold electrodes.

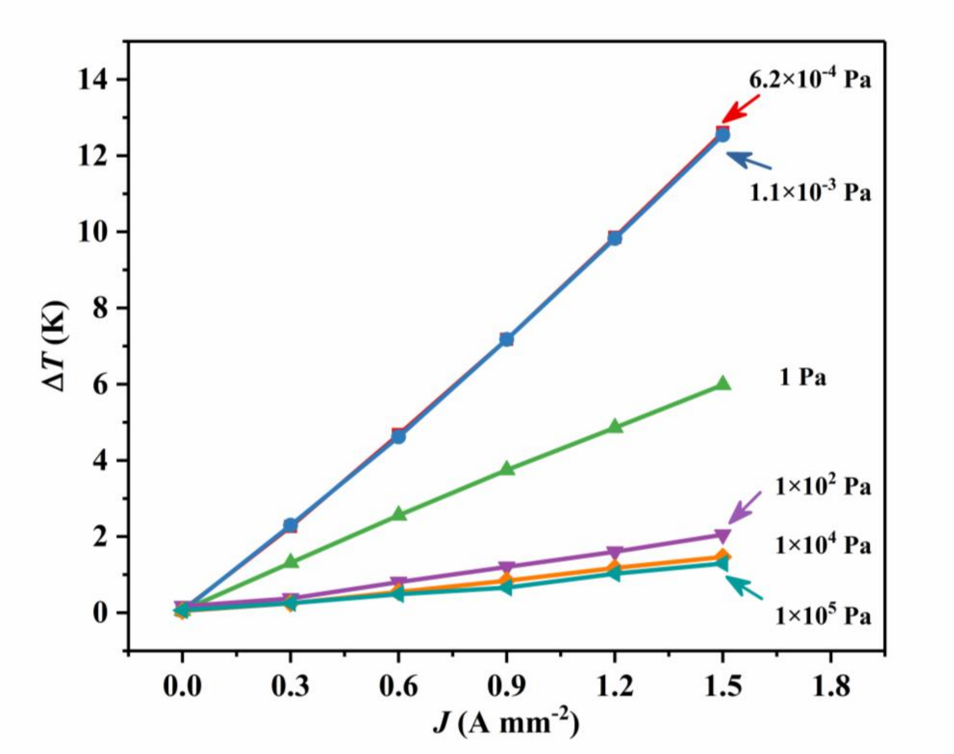

Supplementary Figure 8 | Temperature difference at the two contacts for device measured under different vacuum degrees. The thickness of Parylene substrate is 300 nm.

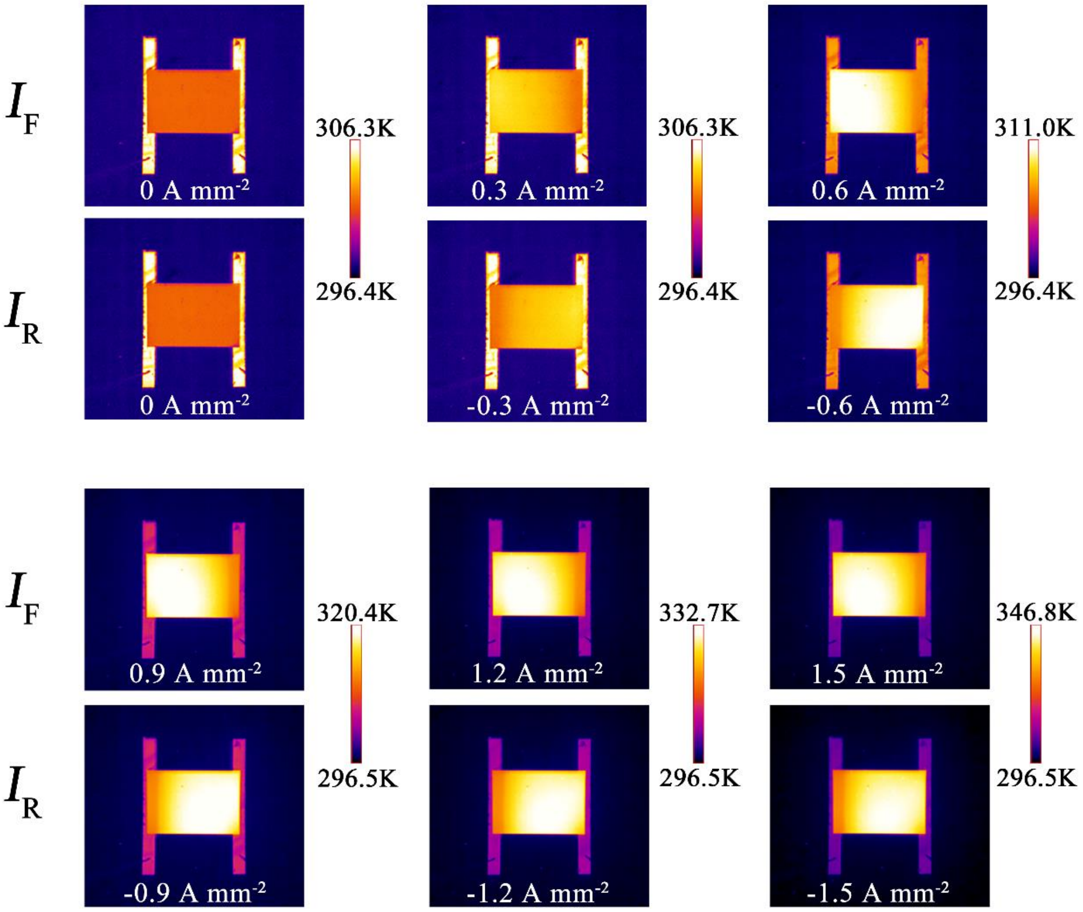

Supplementary Figure 9 | The IR images of a working device at different current densities. I_F and I_R represent the forward and reverse current direction, respectively. The temperature of the exposed electrodes is inaccurate arises from low emissivity of gold electrodes.

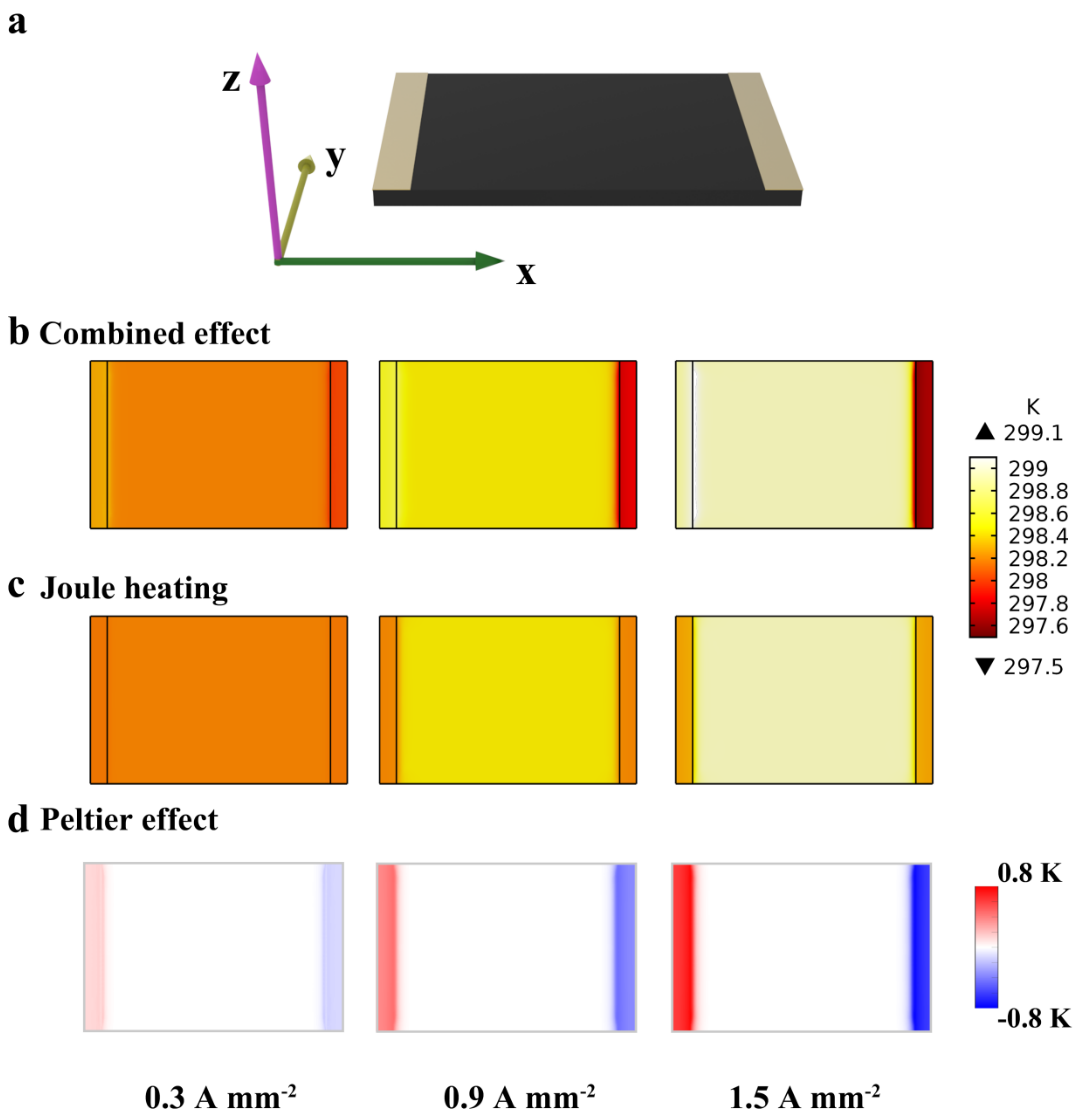

Supplementary Figure 10 | Finite element simulation of a working device. a, Device geometry for finite element analysis. Simulated image of **b,** combined effect, **c,** Joule-heating and **d,** Peltier effect at 0.01 s for various current densities.

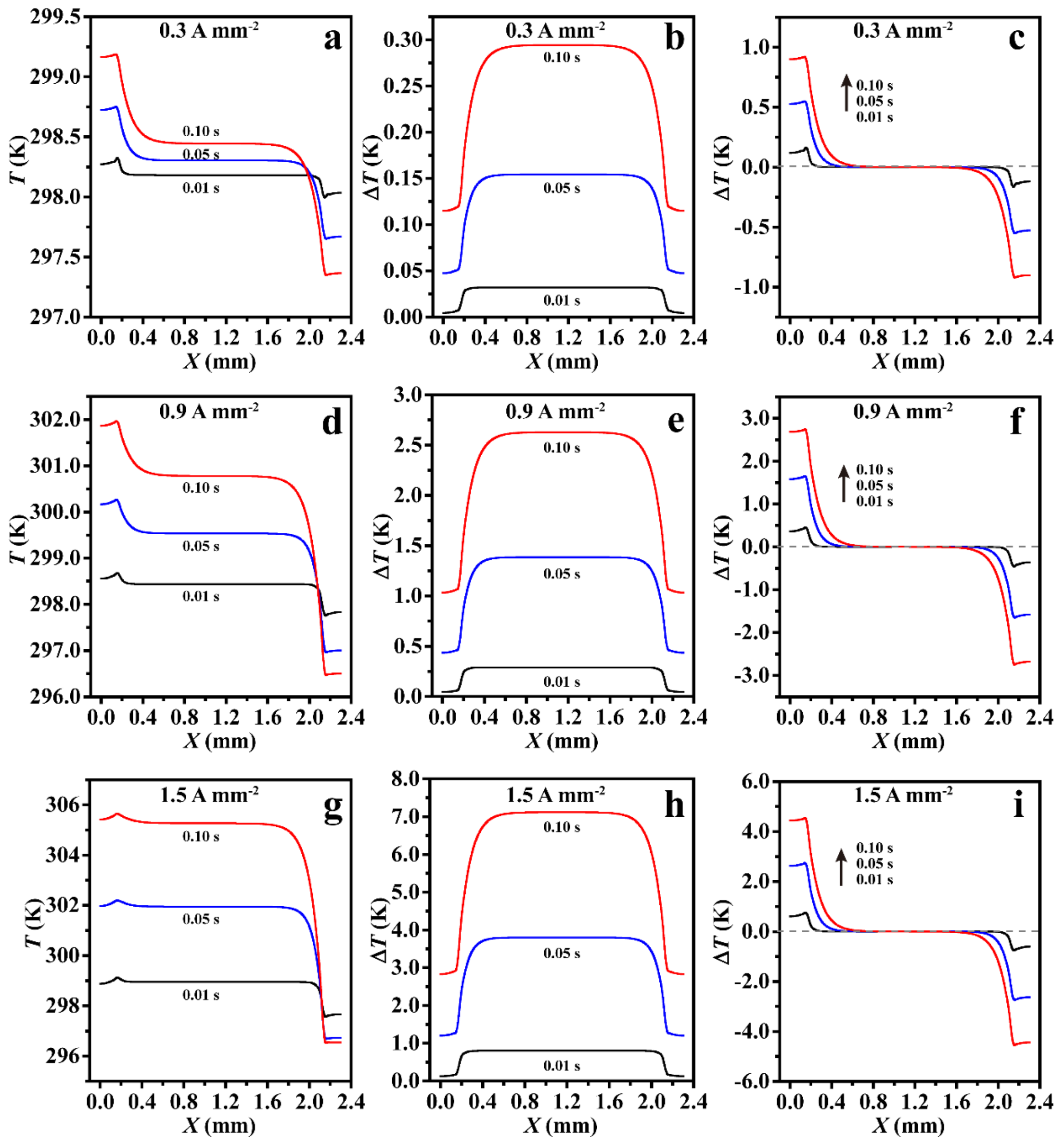

Supplementary Figure 11 | Simulated temperature profile of poly(Ni-ett)-based device. Simulated transient temperature profile contributed by combined effect (**a, d, g**), Joule heating (**b, e, h**) and Peltier effect (**c, f, i**) at current density of **a-b,** 0.3 Amm^{-2}. **d-e,** 0.9 Amm^{-2}. **g-i,** 1.5 Amm^{-2}.

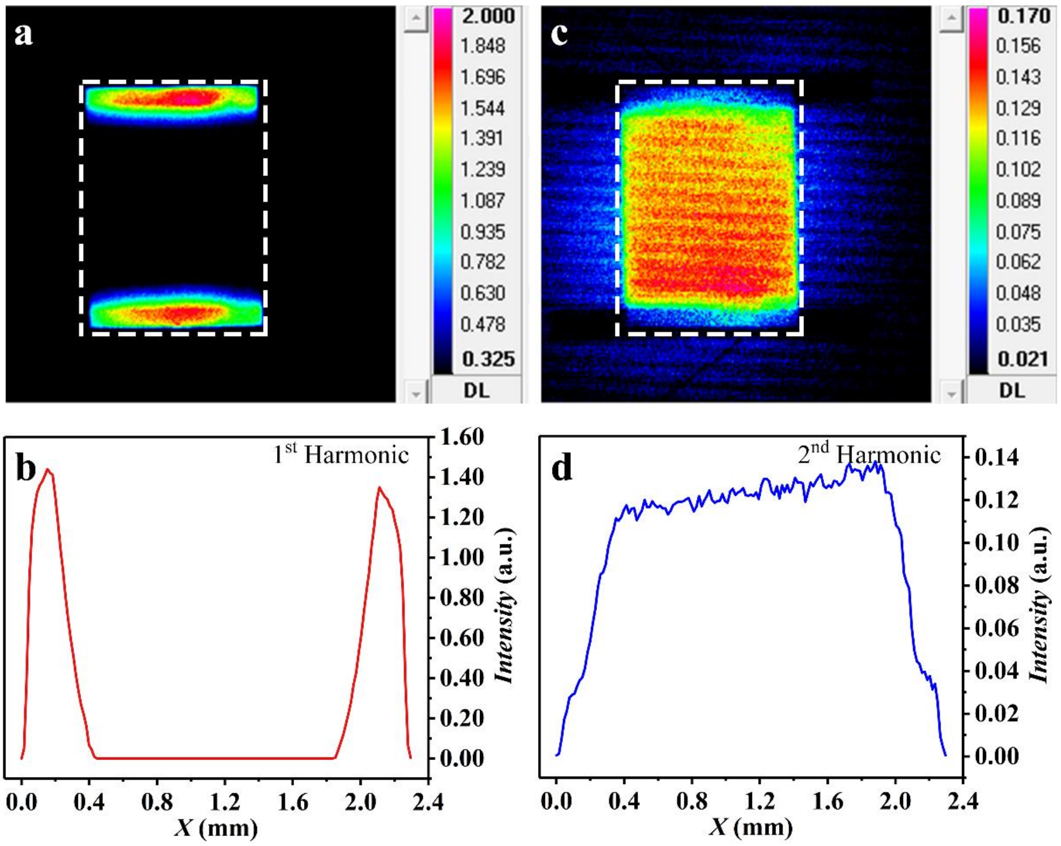

Supplementary Figure 12 | Lock-in thermography measurement. a, Relative amplitude and **b,** relative amplitude profile contributed by Peltier effect. **c,** Relative amplitude and **d,** relative amplitude profile contributed by Joule heating.

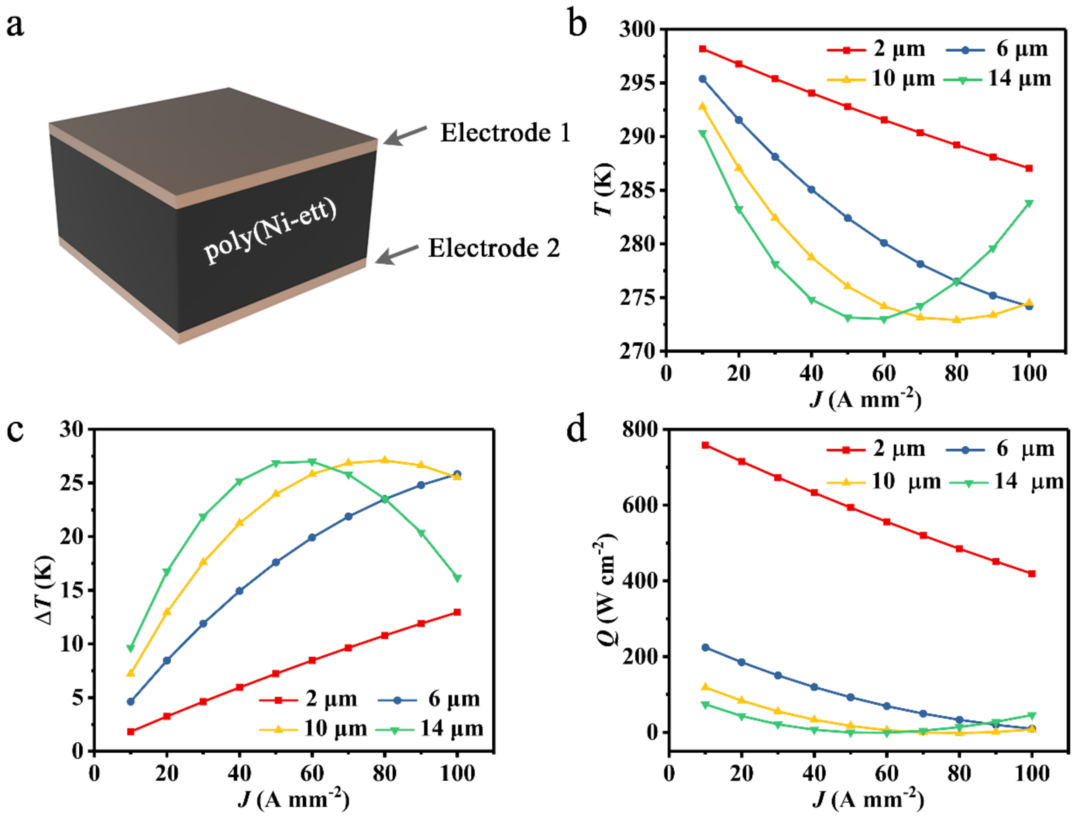

Supplementary Figure 13 | Simulated performance of ultrathin device of poly(Ni-ett). a, Device structure for Peltier effect simulation of ultrathin device. **b,** Cold side temperature of the simulated device. **c,** Temperature difference established by poly(Ni-ett)-based device with different film thicknesses and at current densities. **d,** Heat transport capacity of poly(Ni-ett) based ultrathin device.

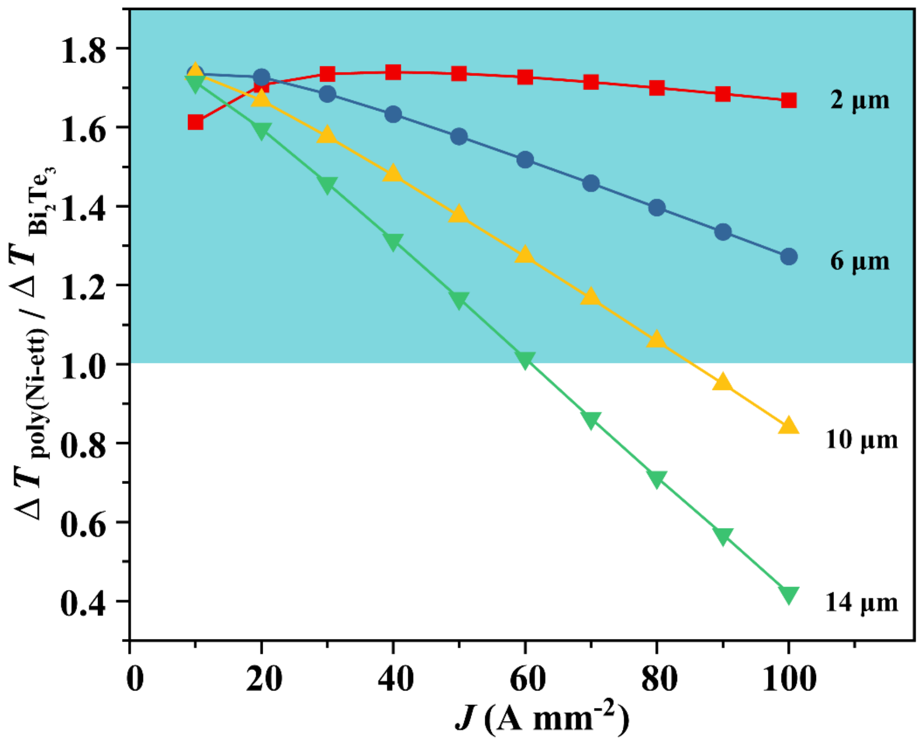

Supplementary Figure 14 | Relative cooling capacity (defined as $\Delta T_{poly(Ni\text{-}ett)}/\Delta T_{Bi2Te3}$) of poly(Ni-ett) and Bi_2Te_3 (simulated by using software built-in parameters) with varied thicknesses and current densities. The cyan square means that the poly(Ni-ett)-based device has advantage in temperature difference maintaining.

Reference

1. Menges, F. et al. Temperature mapping of operating nanoscale devices by scanning probe thermometry. *Nat. Commun.* **7**: 10874 (2016).

2. Grosse, Kyle L. et al. Heterogeneous nanometer-scale Joule and Peltier effects in sub-25 nm thin phase change memory devices. *J. Appl. Phys*. **12**: 124508 (2014).

3. Sun, Y. et al. Flexible n-Type high-Performance thermoelectric thin films of Poly(nickel-ethylenetetrathiolate) prepared by an electrochemical method. *Adv. Mater*. **28**, 3351-3358 (2016).

4. Gao, M. et al. Cooling performance of integrated thermoelectric microcooler. *Solid State Electron*. **43**, 923-929 (1999).
