# Supplementary material for: Exploring Peltier effect in organic thermoelectric films
Source: Nat Commun. 2018 Sep 4;9:3586. doi: 10.1038/s41467-018-05999-4 (PMC6123419; doi:10.1038/s41467-018-05999-4)
Supplement: Supplementary file 3 — Description of Additional Supplementary Files [file 41467_2018_5999_MOESM3_ESM.pdf]

Description of Additional Supplementary Files

File Name: Supplementary Movie 1
Description: Real-time characterization of the temperature differences at the two contacts upon various current biases.
