# Supplementary material for: Exploring Peltier effect in organic thermoelectric films
Source: Nat Commun. 2018 Sep 4;9:3586. doi: 10.1038/s41467-018-05999-4 (PMC6123419; doi:10.1038/s41467-018-05999-4)
Supplement: Supplementary file 2 — Peer Review File [file 41467_2018_5999_MOESM2_ESM.pdf]

Reviewers' comments:

Reviewer #1 (Remarks to the Author):

This paper presents the presence of Peltier effect in organic thermoelectric films. Organic thermoelectric materials received much attention recently. However, to my best knowledge, the Peltier effect, which is commonly observed and particularly applied to temperature controlling systems by using inorganic semiconducting thermoelectric materials, has not been detected in organic thermoelectric films in practice. Thus, this paper will be the first presentation of Peltier effect in organic thermoelectric materials. The experiments are well designed and completed. Thus, this paper can be published in this form. Possibly the following points should be improved before publication.
1. The schematic illustration of the device shown in Figure 1a should be clarified for the readers to correctly and easily understand the structure of the device.
2. The difficulty in the detection of Peltier effect in organic thermoelectric films could be caused by low thermal conductivity of the organic materials. The authors have mentioned on this point in this paper, but should more emphasize this point.

Reviewer #2 (Remarks to the Author):

The manuscript by Zhao, D., et al. utilizes ion gels to create an ionic thermoelectric device with a large thermoelectric voltage. By changing the matrix in which the ionic liquid is swollen, the polarity of the Seebeck coefficient can be changed. While the concept is interesting, this work has many shortcomings in the analysis and conclusions of the data. More rigorous analysis is required by the authors to support the hypotheses made based on the experimental measurements. Furthermore, this work does not provide sufficient substantive advancement or novelty to the current field of organic thermoelectrics (particularly as differentiated from other work by this same research group – specifically references 13 and 25 which include almost all of the same observations of this submission) to justify publication in Nature Materials. Further comments regarding the manuscript are below.

Comments

1. More explanation as to the enhancement of ion transport, first by the fluorinated backbone of PVDF-HFP and hydrogen bonding of PEG, is requested. The authors first state that anions move faster in PVDF-HFP due to repulsive forces from the fluorine groups, but conversely cations move faster in PEG due to attractive forces from the oxygen groups. Having attractive and repulsive forces result in faster ion transport is contradictory.
2. The authors state that PEG molecules interact with TFSI ions through terminal OH groups, leading to slower TFSI diffusion. This is evidenced by the blue shift of the TFSI characteristic mode in the Raman spectrum (Figure 4b). This seems unlikely since there are so few end groups, especially at these low concentrations. At the very least, a test of PEO molecular weight dependence should be done to validate this claim.
3. The authors fabricate a device in which the n-type and p-type ionic thermoelectric materials are connected thermally in parallel and electrically in series. This device is compared to an electronic thermoelectric module, however, this comparison is misleading. In a standard thermoelectric module, n-type and p-type electronic thermoelectrics are connected in series to allow direct current to travel through the circuit, leading to the generation of power. In this study, there is no flow of electrical current, and thus there is no steady state power output.
4. The authors give no justification as to why it is necessary to incorporate n-type and p-type materials in their device. Since there is no charge injection or depletion, it seems that a similar device could have been fabricated using all n-type or all p-type materials with a slightly different electrode configuration. Accordingly, this device is only a minor improvement on the device

reported in reference 13.
5. The materials in this work only present a slight modification to those used in other works (reference 25). While the switch in thermoelectric polarity via PEG addition is interesting, it does not justify a publication in Nature Materials.

Dear Referee,

Thank you very much for the positive evaluations and constructive comments. The manuscript has been revised according to your comments and suggestions. I am uploading the revised version of our manuscript (two copies, in one of them, the changes that we have made are clearly marked in red. The clearly marked version was submitted as related manuscript file). The point-by-point Response to Referees' comments is provided as follows:

Reviewer #1

Comment: This paper presents the presence of Peltier effect in organic thermoelectric films. Organic thermoelectric materials received much attention recently. However, to my best knowledge, the Peltier effect, which is commonly observed and particularly applied to temperature controlling systems by using inorganic semiconducting thermoelectric materials, has not been detected in organic thermoelectric films in practice. Thus, this paper will be the first presentation of Peltier effect in organic thermoelectric materials. The experiments are well designed and completed. Thus, this paper can be published in this form. Possibly the following points should be improved before publication.

Response: We wish to express our sincere thanks to the referee for the encouraging evaluation and constructive comments. We have improved our manuscript according to the comments and suggestions.

Comment #1: The schematic illustration of the device shown in Figure 1a should be clarified for the readers to correctly and easily understand the structure of the device.

Response: Thank you very much for your suggestion. We have modified our schematic illustration of the device to make it clear and easily to understand (Fig. 1a). Moreover, the schematic illustration of a conventional device is moved to the supporting information as the Supplementary Figure S1. The related description has also been improved accordingly. We hope the readers can correctly and easily understand the structure of the device.

Position	Remarks	Revise
**Page 4, Line 18**	The detailed description about the device geometry in Figure 1a has been added.	"The involved thermal processes of a thin-film device with lateral geometry, which is composed by two gold electrodes and the

		poly(Ni-ett) film, are depicted in Fig. 1a."
**Page 5, Line 2-5**	The detailed description about the thermal processes in Figure 1a has been added.	"Along with the Peltier effect, many aforementioned thermal processes also co-exist in an operating device and paly completing role in determining the distribution of temperature. For instance, the joule heating and various heat dissipation processes contribute to the increased and decreased temperature of the device, respectively."
**Page 6, Figure 1**	The caption of Figure 1a has been improved to make it clear and easily to understand.	"Peltier effect, Joule-heating, internal heat transfer within TE film, interlayer heat conduction to the substrate, heat convection to the air, and thermal radiation occurs concurrently in the device."

Comment #2: The difficulty in the detection of Peltier effect in organic thermoelectric films could be caused by low thermal conductivity of the organic materials. The authors have mentioned on this point in this paper, but should more emphasize this point.

Response: We appreciate the reviewer's thoughtful comments. This point has been emphasized in the revised manuscript as follows.

Position	Remarks	Revise
**Page 3, Lines 24**	The difficulty in the detection of Peltier effect in OTE films that caused by low thermal conductivity of the organic materials has	"Precise extraction of the Peltier effect in OTE materials is particularly challenging because of the large contribution of Joule-heating caused by the relative low electrical conductivity

	been emphasized according to the reviewer's suggestion.	of OTE materials[3-7,21], and significant effect of heat dissipation on the measured temperature distribution result from their relative low thermal conductivity."
**Page 5, Lines 12-15**	The difficulty in the detection of Peltier effect in OTE films that caused by low thermal conductivity of the organic materials has been emphasized according to the reviewer's suggestion.	"The result indicates that the relative low thermal conductivity of OTE materials make their temperature distribution susceptible against external atmosphere. Consequently, the detection of Peltier effect in OTE films is challenging and the construction of heat-insulated devices are extremely important for studying the Peltier effect in OTE thin films."

Reviewer #2

Comment: The manuscript by Zhao, D., et al. utilizes ion gels to create an ionic thermoelectric device with a large thermoelectric voltage. By changing the matrix in which the ionic liquid is swollen, the polarity of the Seebeck coefficient can be changed. While the concept is interesting, this work has many shortcomings in the analysis and conclusions of the data. More rigorous analysis is required by the authors to support the hypotheses made based on the experimental measurements. Furthermore, this work does not provide sufficient substantive advancement or novelty to the current field of organic thermoelectrics (particularly as differentiated from other work by this same research group – specifically references 13 and 25 which include almost all of the same observations of this submission) to justify publication in Nature Materials. Further comments regarding the manuscript are below.

1. More explanation as to the enhancement of ion transport, first by the fluorinated

backbone of PVDF-HFP and hydrogen bonding of PEG, is requested. The authors first state that anions move faster in PVDF-HFP due to repulsive forces from the fluorine groups, but conversely cations move faster in PEG due to attractive forces from the oxygen groups. Having attractive and repulsive forces result in faster ion transport is contradictory.

2. The authors state that PEG molecules interact with TFSI ions through terminal OH groups, leading to slower TFSI diffusion. This is evidenced by the blue shift of the TFSI characteristic mode in the Raman spectrum (Figure 4b). This seems unlikely since there are so few end groups, especially at these low concentrations. At the very least, a test of PEO molecular weight dependence should be done to validate this claim.

3. The authors fabricate a device in which the n-type and p-type ionic thermoelectric materials are connected thermally in parallel and electrically in series. This device is compared to an electronic thermoelectric module, however, this comparison is misleading. In a standard thermoelectric module, n-type and p-type electronic thermoelectrics are connected in series to allow direct current to travel through the circuit, leading to the generation of power. In this study, there is no flow of electrical current, and thus there is no steady state power output.

4. The authors give no justification as to why it is necessary to incorporate n-type and p-type materials in their device. Since there is no charge injection or depletion, it seems that a similar device could have been fabricated using all n-type or all p-type materials with a slightly different electrode configuration. Accordingly, this device is only a minor improvement on the device reported in reference 13.

5. The materials in this work only present a slight modification to those used in other works (reference 25). While the switch in thermoelectric polarity via PEG addition is interesting, it does not justify a publication in Nature Materials.

Response: Many thanks for the review. We find these comments are not related to our manuscript. In our manuscript, we demonstrate the experimentally observation and quasi-quantitative evaluation of the Peltier effect in OTE materials. However, these comments are related to the utilization of ion gels to create an ionic thermoelectric device with a large thermoelectric voltage. Moreover, the mentioned author (Zhao, D) in the comment is not listed in our manuscript. It seems that these comments are related to a manuscript submitted to *Nat. Mater.* rather than our manuscript submitted to *Nat. Commun.* As a result, we have no responses to these comments although we

appreciate the referee’s effects in reviewing our manuscript.

REVIEWERS' COMMENTS:

Reviewer #1 (Remarks to the Author):

The manuscript on Peltier effect by Wenlong Jin et al. has been a little improved. The revised manuscript can be published in Nature Communication in the present form.
